# Matching the opposites: liver transplantation from a situs viscerum inversus totalis donor

**DOI:** 10.1007/s13304-024-01968-3

**Published:** 2024-11-13

**Authors:** Cristiano Guidetti, Roberta Odorizzi, Barbara Catellani, Philip Muller, Paolo Magistri, Gian Piero Guerrini, Stefano Di Sandro, Fabrizio Di Benedetto

**Affiliations:** 1 Hepato-Pancreato-Biliary Surgery and Liver Transplantation Unit, University Hospital of Modena “Policlinico”, University of Modena and Reggio Emilia, 41124 Modena, Italy; 2 Clarunis – University Centre for Gastrointestinal and Hepatopancreatobiliary Diseases, Basel, Switzerland

**Keywords:** Situs viscerum inversus totalis, Liver transplantation, Liver procurement

## Abstract

Situs viscerum inversus totalis (SIT) is a rare congenital anomaly. Deceased donors with this condition are often declined because of the technical issues in both the organ’s procurement and its transplant. Only eight cases of deceased donor organs with SIT were reported to be used for liver transplantation (LT). We herein present a case of LT using a graft from an SIT donor: a modified retroversus piggyback technique was used. A 15 year-old female was referred to our institution as a potential donor. An SIT condition was discovered during standard donor evaluation together with the presence of a complex triple arterial pedicle. Procurement operative time was 125 min, from skin incision to cross-clamp. Liver extraction occurred 32 min after cold flush. The recipient was a 56 year-old male affected by recurrent hepatocellular carcinoma (HCC) on hepatitis C related liver cirrhosis. Position and orientation trials of the graft were made and it was decided to implant it with the retroversus technique. Direct duct-to-duct biliary reconstruction was achieved. The postoperative course was uneventful. To our knowledge, this is the first implant with retroversus technique combined to direct biliary reconstruction and the first repetition of that technique. Cases like this highlight how technical complexity can be overcome leading to successful management of difficult scenarios in a safe manner.

## Introduction

Situs viscerum inversus (SVI) is a broad spectrum of anatomical variation that is found in 0.01% of the general population [1].

SVI totalis (SIT) is even less frequent (1 out of 8000) and defined when the whole body anatomy is on the opposite side of normal disposition, involving both thoracic and abdominal organs. Many pathological conditions can be associated with this including agenesia of the inferior vena cava and biliary atresia [2].

Organ procurement from donors with SVI is a demanding surgical procedure and has been very rarely described. Thoracic organs such as lungs and heart are generally not considered for transplantation due to the difficult matching with the recipient, while, on the other hand, reports of SIT patients transplanted with regular thoracic organs are available [3].

For the abdominal organs, kidneys from SIT donors do not arouse concerns, while liver transplantation (LT) can be hazardous because of the complex implantation with the different anatomy.

In the few reported cases of LT from SIT donors, different implantation techniques were described. One option is standard piggyback implantation [4], and on the other hand, the so-called retroversus technique can be adopted. In this last case, a 180° rotation on the cranio/caudal axis is performed allowing a reduction in graft mobility due to its better fit in the right fossa, allowing the prevention of kinking and outflow obstruction [5].

We herein present a case of organ procurement and LT using a graft from an SIT donor using retroversus technique.

## Case report

A 15-year-old female was referred to our institution as a potential donor. The cause of brain death was a cerebrovascular accident due to rupture of an arterio-venous malformation.

An SIT condition was discovered during standard donor evaluation with a thoraco-abdominal computed tomography scan (CT). Liver function tests were within normality range and viral hepatitis was excluded by serology.

The CT angiography demonstrated the presence of a complex triple arterial pedicle composed of a proper hepatic artery from the celiac trunk, a replaced artery from the gastric artery and a second replaced artery from the superior mesenteric artery. Portal vein was regularly bifurcated and its course was standard. No biliary duct anatomy variants were identified.

Procurement operative time was 125 min, from skin incision to cross-clamp. Liver extraction occurred 32 min after cold flush and perfusion; standard static cold storage was set for transportation.

**Fig. 1:**
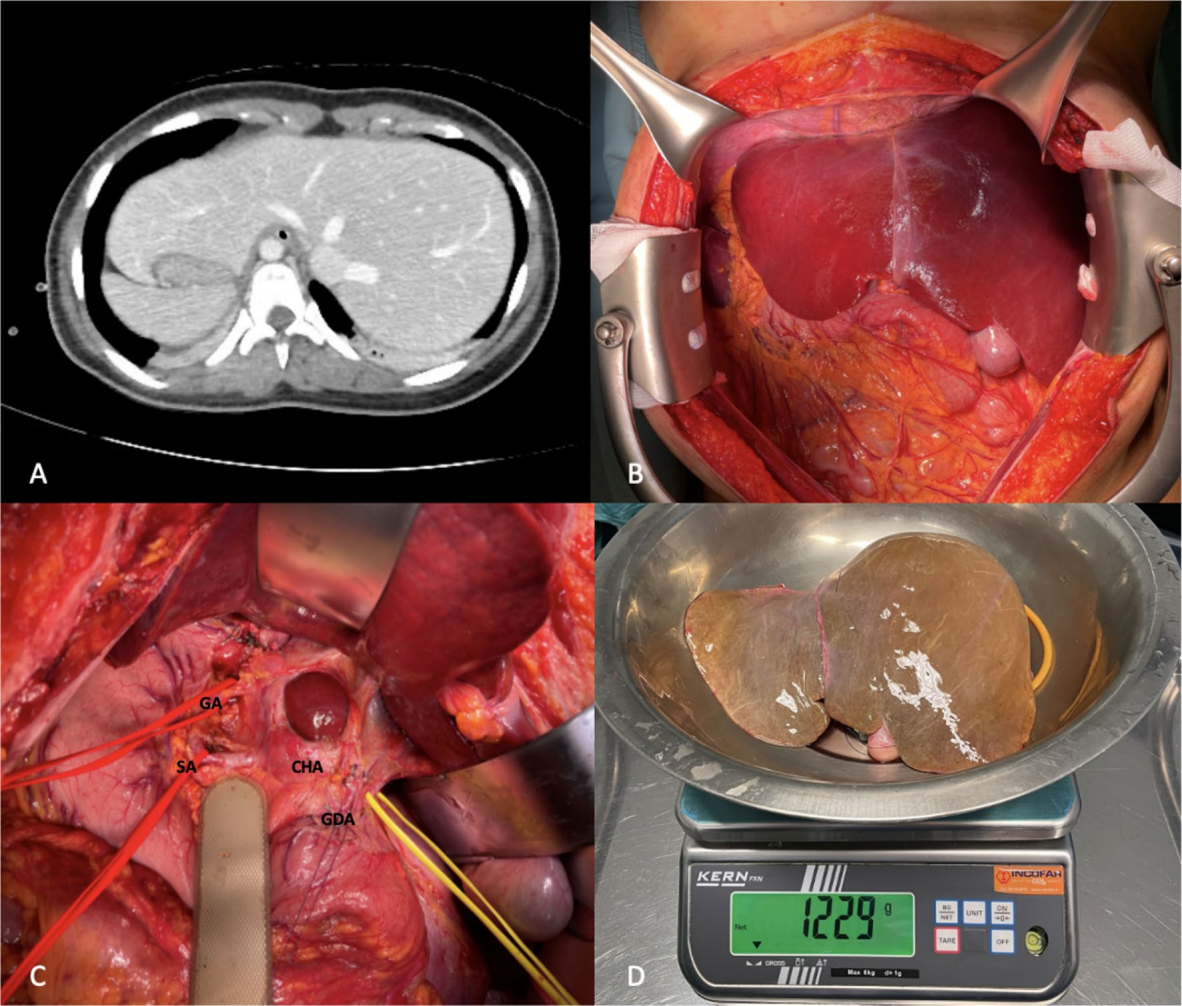
A, CT scan of the donor at the moment of procurement; B, Macroscopic appearance of the liver; C, Preparation of the hepatic hilum, gastric (GA) and splenic artery (SA) on red vessel loops, common biliary duct on yellow vessel loop, gastroduodenal artery (GDA) on violet tie and common hepatic artery (CHA); D, Harvested graft after flush

During procurement, no technical issues occurred and liver biopsy was not performed, since the liver had normal macroscopic appearance and no previous liver disease was present in this young donor. Details of the procurement are displayed in Fig. 1.

The selected recipient was a 56-year-old male patient affected by recurrent hepatocellular carcinoma (HCC) on hepatitis C-related liver cirrhosis. He was already treated with a trans-arterial chemoembolization (TACE) as a bridge to LT. MELD score was 11, CPT A6, and waiting list time 60 days. His vascular anatomy was normal.

Liver graft was prepared at the bench, reconstructing the accessory artery from the superior mesenteric artery, which was passed anteriorly to the portal vein, on to the gastroduodenal artery (GDA) to create a single pedicle for implantation on the celiac trunk.

Liver graft weight was 1229 g and graft to body weight ratio 1,75.

Hockey stick incision was performed and hepatectomy was conducted with accurate preservation of the inferior vena cava. Hepato-caval confluence was clamped and hepatectomy completed.

Position and orientation trials with the liver graft were made at this time. We decided for the retroversus technique, creating a caval anastomosis between the suprahepatic vena cava of the graft and the adapted cuff of right and middle/left hepatic vein, using continuous 4–0 Prolene sutures. The portal vein was reconstructed with an end-to-end anastomosis using 6–0 Prolene.

Total cold ischemia time was 6 h and 2 min. After portal reperfusion of the graft, the celiac trunk of the donor was implanted on the proper hepatic artery of the recipient at the GDA branch patch with a running suture in Prolene 7–0. Direct duct-to-duct biliary reconstruction was achieved with continuous suture in Maxon 6–0. The total operative time was 5 h and 40 min and no intraoperative transfusions were necessary.

**Fig. 2:**
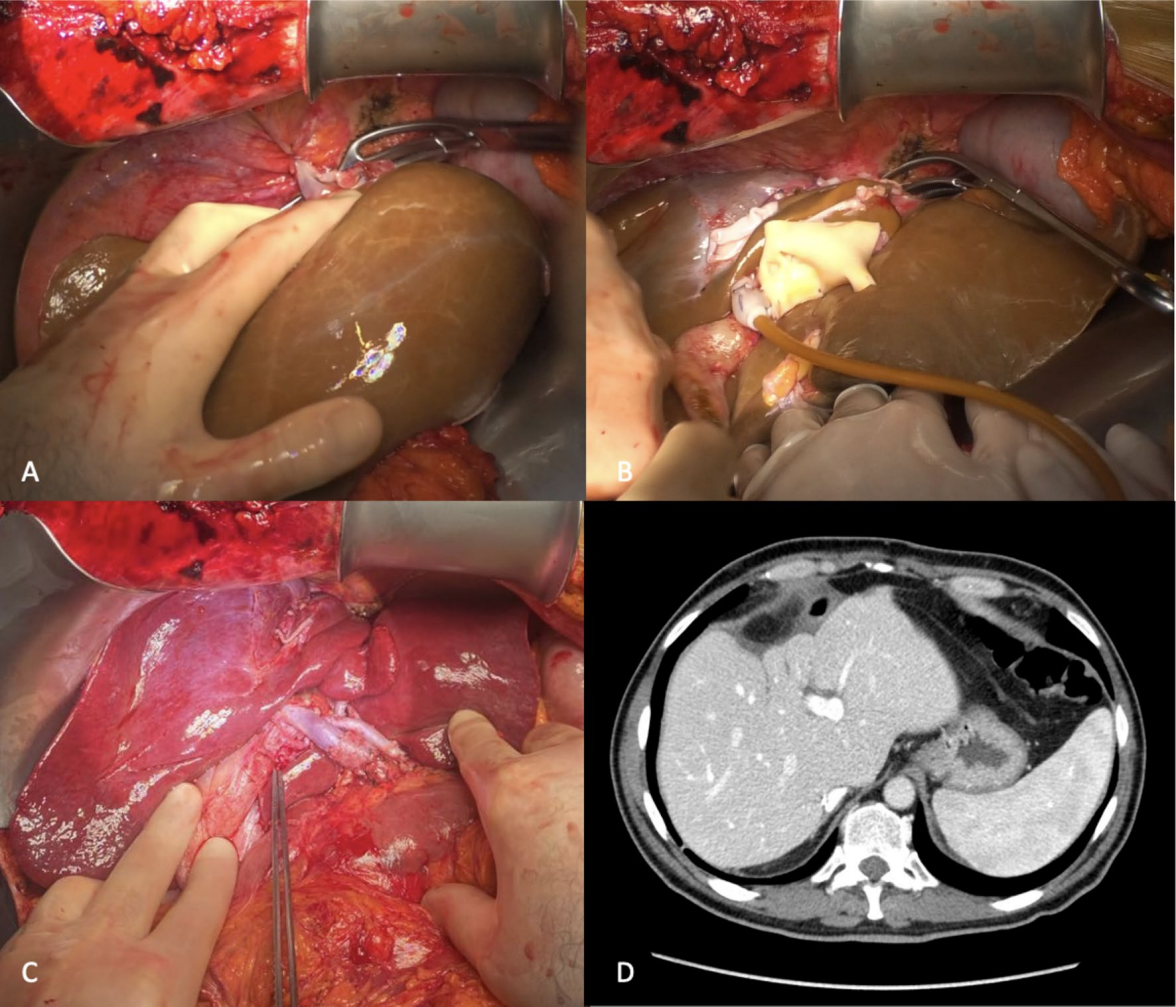
A, Trial of positioning the graft in a standard fashion; B, Preparation for implant using retroversus technique; C, Surgical field after reperfusion of the graft and arterial reconstruction; D, CT scan of the recipient 45 days after LT

**Fig. 3:**
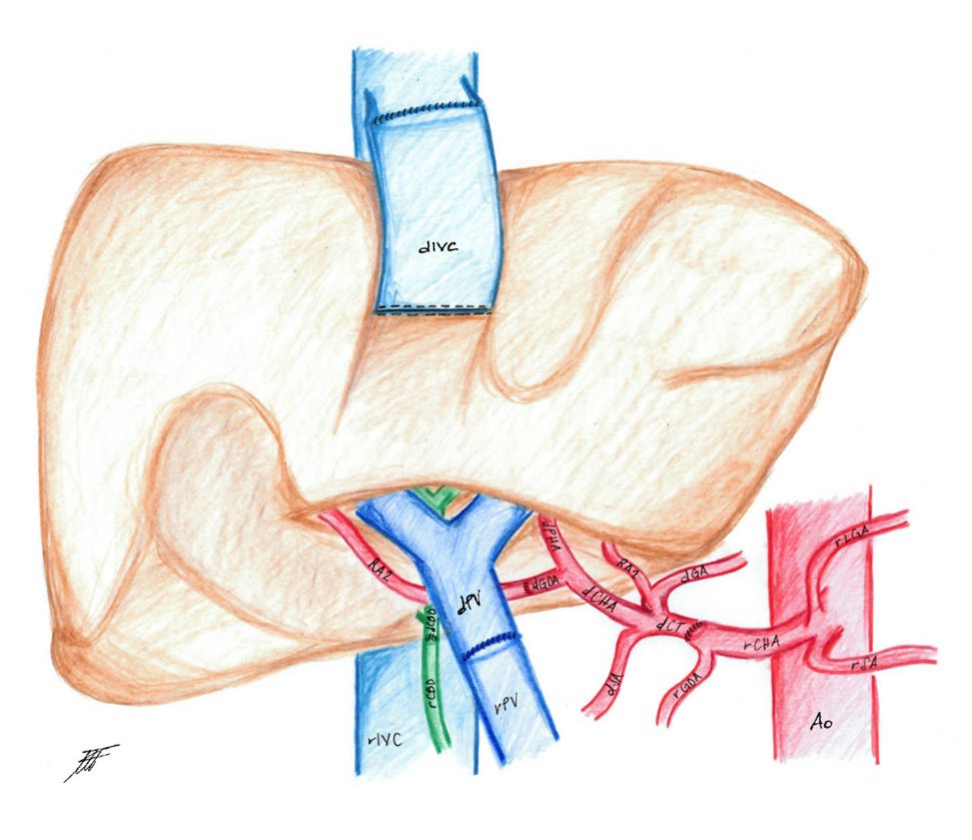
Schematic rappresentation of the implant technique; Aorta (Ao); recipient splenic artery (rSA); recipient left gastric artery (rLGA); recipient common hepatic artery (rCHA); recipient gastroduodenal artery (rGDA); donor celiac trunk (dCT); donor gastric artery (dGA); replaced artery from gastric artery (RA1); donor splenic artery (dSA); donor common hepatic artery (dCHA); donor proper hepatic artery (dPHA); donor gastroduodenal artery (dGDA); replaced artery arising from donor superior mesenteric artery anastomosed with dGDA (RA2); recipient portal vein (rPV); donor portal vein (dPV); donor inferior vena cava (dIVC); recipient inferior vena cava (rIVC); donor common bile duct (dCBD); recipient common bile duct (rCBD)

Details of the transplant are displayed in Fig. 2 and its schematic representation in Fig. 3.

The postoperative course was uneventful. Immunotolerance induction therapy was administered with basiliximab 20 mg at the time of transplant and on POD 4. Standard immunosuppressive regimen with FK506 was prescribed, maintaining hematic concentration between 7 and 9 ng/ml.

The ICU stay was 24 h and the patient was discharged from hospital on POD 7. Surveillance CT scan was performed on postoperative day 45 showing regular surgical outcomes; particularly no anastomotic issues were identified.

After 15 months, the patient is in optimal general conditions, with LFTs within normal range.

## Discussion

This case triggered an intensive technical discussion before LT in our unit and several key steps should be highlighted.

Before procurement, triphasic CT scan of the donor is key to accurately plan the surgical procedure, which should be performed by an experienced procurement surgeon. Beyond confirming the SIT diagnosis, preoperative multiphase CT allows identification of hepatic arterial vascularization variants that have to be preserved at the time of the procurement. Performing the procurement from the left side of the patients may help in maintaining standard direction of the bilio-vascular structures, but may require higher dexterity with the left hand. The side of approach is on surgeon’s preference and may change secondary to the phase of the procurement.

As in normal LT, back bench preparation should focus on preparing the longest possible pedicle to allow all possible reconstructions. In our opinion, preoperative decision-making on the specific surgical technique is detrimental. To keep an open mind toward different possible solutions is key to perform a safe implantation. The team should meet and discuss, prior to the beginning of the case, all possible strategies of reconstruction. Prosthetics material such as PTFE grafts and bovine pericardium patches may be helpful tools in case of necessity to elongate vessels to prevent position-related occlusions. Particularly, attention to twisting and kinking of the vascular pedicles is fundamental to obtain good outcomes.

After completion of the hepatectomy, we strongly advise for position trials before deciding on the implantation technique to be used. During the attempts, the graft should be protected by icy wet sponges to avoid possible negative impact of longer warm ischemia.

The choice of the approach depends on both the donor’s and recipient’s characteristics.

In our opinion, large-size recipients allow for more facile allocation of the graft. On the other hand, smaller or previously operated recipients may have limited space forcing the orientation of the graft. Also, the graft itself, if large in size, may prevent the use of retroversus technique, since thick dominant lobes may increase the distance between the vena cava of the graft and the donor.

In our opinion, if in the retroversus position the graft allocates smoothly in the right upper quadrant with adequate distance between the vascular structures, this is the technique of choice. Standard piggyback technique may facilitate caval anastomosis, but leaves the graft’s greatest lobe in the left quadrant creating bulging in that region and an empty space toward the right side that can increase the risk of dislocation and subsequent torsion/kinking of the vessels.

Attention to both inflow and outflow is key to prevent a difficult situation: in the study of Reimondez et al., they started with a retroversus approach and had to switch to a standard implant because of inadequate length of the portal vein [6]. On the other hand, excessive length of portal reconstruction should be avoided to minimize the mentioned risks.

In our case, after position trials, we opted for the retroversus technique which allowed us a good fit of the liver under the right rib cage without compromising the possibility of a straight inflow reconstruction. Portal vein was the most anterior structure of the reconstructed hilum, in front of the arterial pedicle. Direct bile duct reconstruction was possible without any tension to the common bile duct of the recipient.

To our knowledge, this is the first implant with retroversus technique combined with direct biliary reconstruction and the first repetition of the technique described by Pomposelli et al [5].

As displayed in Table 1, reported literature in this setting is extremely scarce and does not highlight evidence that a technique is better than the other. Actually, tailored approach is the best option in these circumstances [7]. Only eight cases of liver transplants from SIT donors have been reported before this. No other author has highlighted the necessity of a fit trial, but we consider this key to safe and successful transplant.

**Table 1:** Reported cases of transplant from SIT donors

Author	Arterial anatomy of the graft	Technique	Caval anastomosis	Biliary reconstruction	Positioning trial reporting	Outcome
Asfar (1995)	Accessory hepatic artery from gastric artery	Piggyback	End to side with graft’s infrahepatic vena cava	Hepatico-jejunostomy	No	died on POD 20 due to sepsis and biliary leak
Herrera (1996)	Modal	Piggyback	End to side	Duct to duct	No	Alive 30 months
Braun (1998)	Modal	Piggyback	End to side	Duct to duct	No	Alive 17 months
Pomposelli (2007)	Replaced hepatic artery from superior mesenteric artery	Piggyback	Retroversus technique (end to side)	Hepatico-jejunostomy	No	Non occlusive clot in inferior vena cava, no long term follow up
Dou (2010)	Modal	Classic technique	End to end (tissue expander to prevent rotation)	Duct to duct	No	Alive 10 months
Sun (2013)	Modal	Piggyback	End to side	Duct to duct	No	Alive 36 months
Manzia (2014)	Modal	Piggyback	Side to side	Duct to duct	No	Alive 6 months
Reimondez (2018)	Modal	Piggyback	End to end	Hepatico-jejunostomy	Yes, retroversus technique attempted and then undone in favor of standard implant	No long term follow up
Guidetti (2024, present case)	Accessory from gastric artery and accessory from superior mesenteric artery	Piggyback	Retroversus technique (end to side)	Duct to duct	Yes, retroversus implant chosen because best fit	Alive 10 months

There is no technical limit, since cases of donor hepatectomy for LDLT have been reported in donors with SIT [8]. Differently from LDLTs, accurate preoperative planning including 3D reconstruction is generally not possible in the setting of deceased donors. For these reasons, such cases may benefit from the expertise of high-volume centers that are used to manage complex vascular reconstruction and have active programs of adult-to-adult split liver and/or living donor liver transplant. Moreover, we took inspiration for this reconstruction from the experience of pediatric transplant with monosegment grafts that frequently require to be flipped to get positioned in the right hypochondrium [9].

Given the low incidence of this condition, an international effort to collect data on this topic is desirable to obtain more insight into its management.

In conclusion, cases like this highlight how technical complexity can be overcome leading to successful management of difficult scenarios in a safe manner.

## Data Availability

Data regarding this case report are available from the corresponding author upon request.
